# Analysis of coelom development in the sea urchin *Holopneustes purpurescens* yielding a deuterostome body plan

**DOI:** 10.1242/bio.015925

**Published:** 2016-02-18

**Authors:** Valerie B. Morris

**Affiliations:** School of Biological Sciences A12, University of Sydney, New South Wales 2006, Australia

**Keywords:** Ontogeny, Evolution, Homology, Pentamery, Growth-zone, Metamerism

## Abstract

An analysis of early coelom development in the echinoid *Holopneustes purpurescens* yields a deuterostome body plan that explains the disparity between the pentameral plan of echinoderms and the bilateral plans of chordates and hemichordates, the three major phyla of the monophyletic deuterostomes. The analysis shows an early separation into a medial hydrocoele and lateral coelomic mesoderm with an enteric channel between them before the hydrocoele forms the pentameral plan of five primary podia. The deuterostome body plan thus has a single axial or medial coelom and a pair of lateral coeloms, all surrounding an enteric channel, the gut channel. Applied to the phyla, the medial coelom is the hydrocoele in echinoderms, the notochord in chordates and the proboscis coelom in hemichordates: the lateral coeloms are the coelomic mesoderm in echinoderms, the paraxial mesoderm in chordates and the lateral coeloms in hemichordates. The plan fits frog and chick development and the echinoderm fossil record, and predicts genes involved in coelomogenesis as the source of deuterostome macroevolution.

## Abstract (precis)

**Summary:** A common body plan for echinoderms, chordates and hemichordates resolves the apparent morphological disparity between the pentameral and the bilateral body plans of these major deuterostome phyla.

## INTRODUCTION

Deuterostomes are a supraphyletic group comprising three major phyla, the echinoderms, the chordates and the hemichordates. Molecular phylogenetic evidence supports a monophyletic origin for the group (Putnam et al., 2008). Morphologically, however, the three major phyla are problematic since the pentameral body plan of echinoderms seems to be quite different from the bilateral body plans of chordates and hemichordates. Here, the fundamental morphological homology between the pentameral and the bilateral body plans is identified in the derivation of a body plan for deuterostomes.

The body plan of echinoderms is in general pentamerous, that is to say it is made up of five arms or rays and its structure and evolutionary origin are better understood when treated as a pentameral body plan rather than, as it is often described, a radial plan. Traditionally, there is a further division into five ambulacra separated by five interambulacra. The ambulacra are the radial canals and tube feet and the interambulacra are the plates and tissues between them (Hyman, 1955). More recently, Mooi et al. (1994) referred back to Jackson who in 1912 described each arm or ray as a set of four columns of plates: the two central columns were ambulacral columns and each outer column was a single interambulacral column. Each interambulacral region was thus split into two. The columns of four plates grow from a zone at the tips of the arms or rays that is referred to as Jackson's growth zone. It is this unit of four columns of plates and Jackson's growth zone that is key to understanding the pentameral body plan of echinoderms. This is so, first because the pentameral body plan can be explained by duplications of the four columns of plates arising from one Jackson's growth zone (Morris, 2012). Duplications are in agreement with the fossil record of the co-existence of bilateral and three and five-rayed echinoderms during the Cambrian (Smith et al., 2013). Then secondly, accepting a hypothesis of duplication, morphological homology between the echinoderm, hemichordate and chordate body plans will be, predictably, in the similarities between the structures of one echinoderm arm or ray and the anterior-posterior axial structures of hemichordates and chordates.

The morphological homology of the deuterostome phyla was approached in the past using similarities between larval forms and similarities in the arrangement of coeloms, as well as by applying characters common to, or characteristic of the phyla. The writings are an intermix of what the ancestor was like and what the path of evolution might have been. Bather (1900) described a hypothetical bilateral larva, the dipleurula larva, as the ancestral larva of echinoderms while Bury (1895) described a pentactula larval ancestor with five tentacles around the mouth. Similarities between echinoderm and hemichordate larvae had earlier led to a link between the echinoderms and the hemichordates and the name, the Ambulacraria (Hyman, 1955, p2). The origin of chordates from echinoderms was explained by Jefferies (1986) as dexiothetism, that the ancestral larva lay on its right side leading to a reduction in right side structures. The chordate nervous system was derived by Garstang (1928) from larval ciliated bands, an idea now re-assessed by Lacalli (2005). The three-part division of larval coeloms into axocoele, hydrocoele and somatocoele, originally named by Heider (1912), was envisioned by Peterson et al. (2000) as the arrangement of coeloms in the common ancestor of hemichordates and echinoderms, with the arms of echinoderms regarded as outgrowths from an echinoderm anterior-posterior axis identified by gene expression. The idea that the three coeloms were oligomerous segmentation had earlier been rejected by Hyman (1955, p692). Minsuk et al. (2009) derived two axes for echinoderms, a circum-oral axis of pentamery and a proximo-distal axis of structure along the arms. Characters prominently used in describing the origins of vertebrates have been reviewed by Swalla (2007). Satoh et al. (2012) have reviewed the origin of the chordate notochord, implicating a new expression domain for the T-box gene, *Brachyury*. Gislén (1930), who proposed the general terms protocoele, mesocoele and metacoele for the three-part division of larval coeloms, regarded the echinoderm hydrocoele and the chordate notochord as homologous. The transition from bilateral symmetry to pentaradiality has been reviewed by Smith et al. (2004). Raff and Popodi (1996) raised the possibility that pentamery was a consequence of duplication of the echinoderm hydrocoele. Hotchkiss (1998) proposed a balanced duplication for the five echinoderm arms. Given little attention previously, the secondary podia of the water-vascular system of echinoderms have been described recently as a serial repetition different from metamerism by David and Mooi (2014), but as metamerism, a metazoan character, by Morris (2009, 2012).

The deuterostome body plan is derived from ontogenetic evidence extracted from a further analysis of the morphological development of coeloms in the sea urchin *Holopneustes purpurescens* (Agassiz, 1872). This sea urchin develops directly, progressing from gastrulation to the juvenile sea urchin without an intervening, feeding larval stage (Morris, 1995). The coeloms that develop in this sea urchin are those of the adult sea urchin. Two earlier descriptions of the development of coeloms in *H. purpurescens* identified a mouth opening (Morris, 2007) and an archenteron opening (Morris, 2012), which are parts of the enteric channel described here. Duplication of rays and metamerism were proposed as characters of the echinoderm pentameral body plan and thence homology with the chordate body plan (Morris, 2012).

Here, the morphological homology between the three major deuterostome phyla derived from the further interpretation of adult coelom development in *H. purpurescens* is encapsulated in the deuterostome body plan. Essential findings are the transition of the anterior-posterior axis to an oral-aboral axis early in the development of the coeloms and the existence of an enteric channel that arises from the archenteron and whose track follows the changed direction of the body axis. Importantly, three coeloms develop from the walls of the archenteron: the medial hydrocoele forms from the aboral wall of the archenteron while coelomic mesoderm forms on each side of the archenteron from its lateral walls. The primary podia develop from the hydrocoele in a group of three and a group of two. The distinguishing feature of the third, medial coelom that echinoderms share with chordates and hemichordates is an addition to the paired coeloms of protostomes and is possibly a deuterostome invention.

## RESULTS

**Fig. 1.**
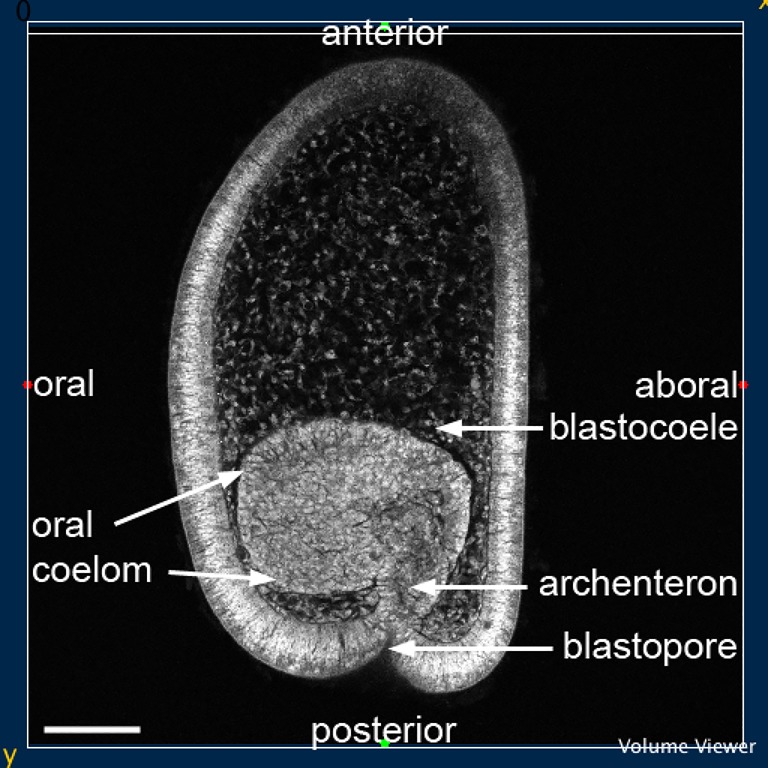
**Larva of *H. purpurescens* at 29 h**. Left-side sagittal view showing the oral coelom, archenteron and blastopore and the anterior, posterior, oral and aboral adult orientation (refer to text). Scale bar: 100 µm.

**Fig. 2.**
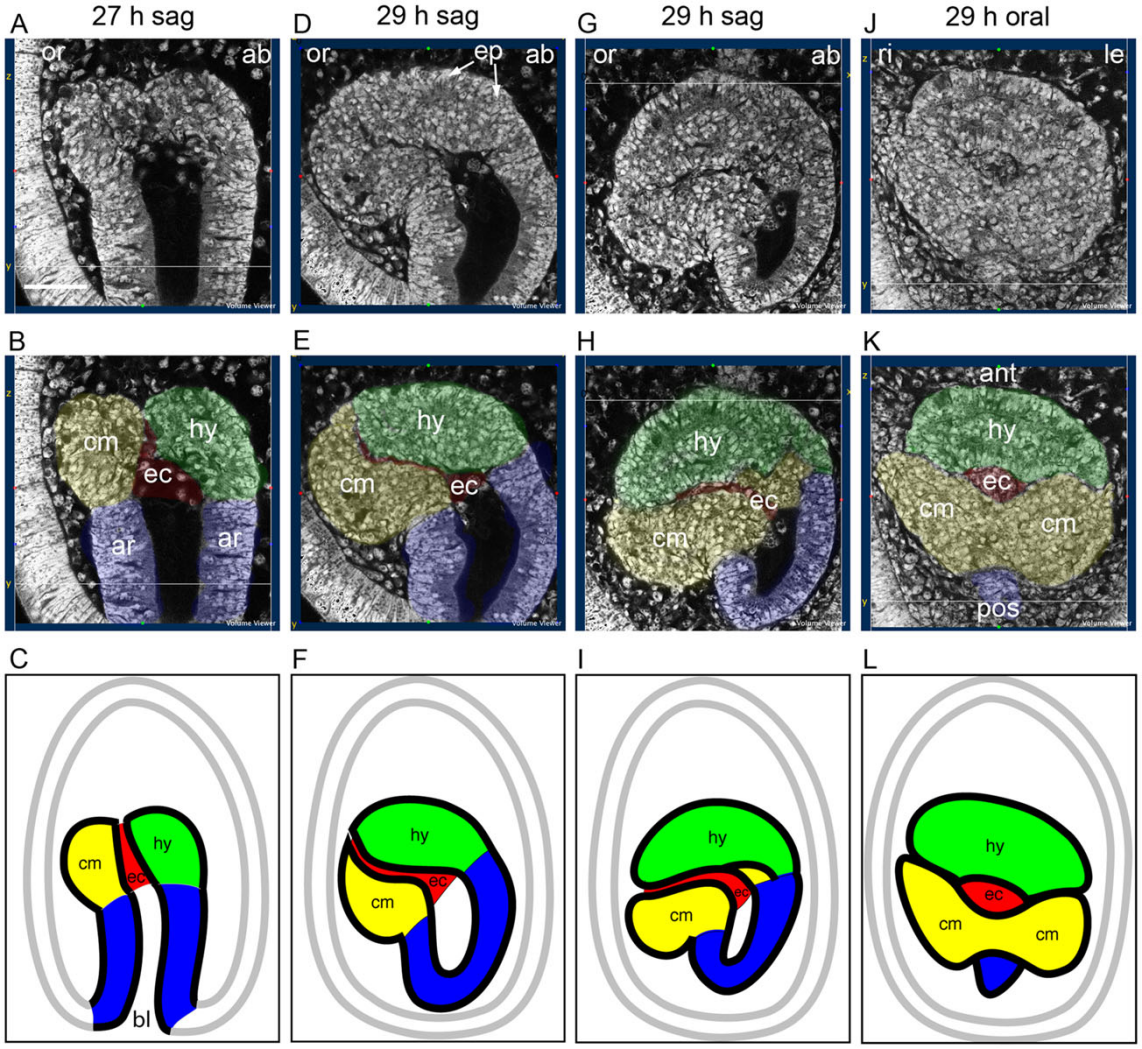
**Early development of the coeloms and the enteric channel.** (A,D,G,J) Sections of larvae at 27 h and 29 h, uncoloured. (B,E,H,K) The same sections coloured with tissues of the hydrocoele (hy) green, coelomic mesoderm (cm) yellow, enteric channel (ec) red and archenteron (ar) blue. (C,F,I,L) Schematics of the sections with the tissues coloured similarly. A-I, sagittal views; J-L, oral view. ab, aboral; ant, anterior; bl, blastopore; ep, hydrocoele epithelium; le, left; or, oral; pos, posterior; ri, right. Scale bar: 50 µm.

**Fig. 3.**
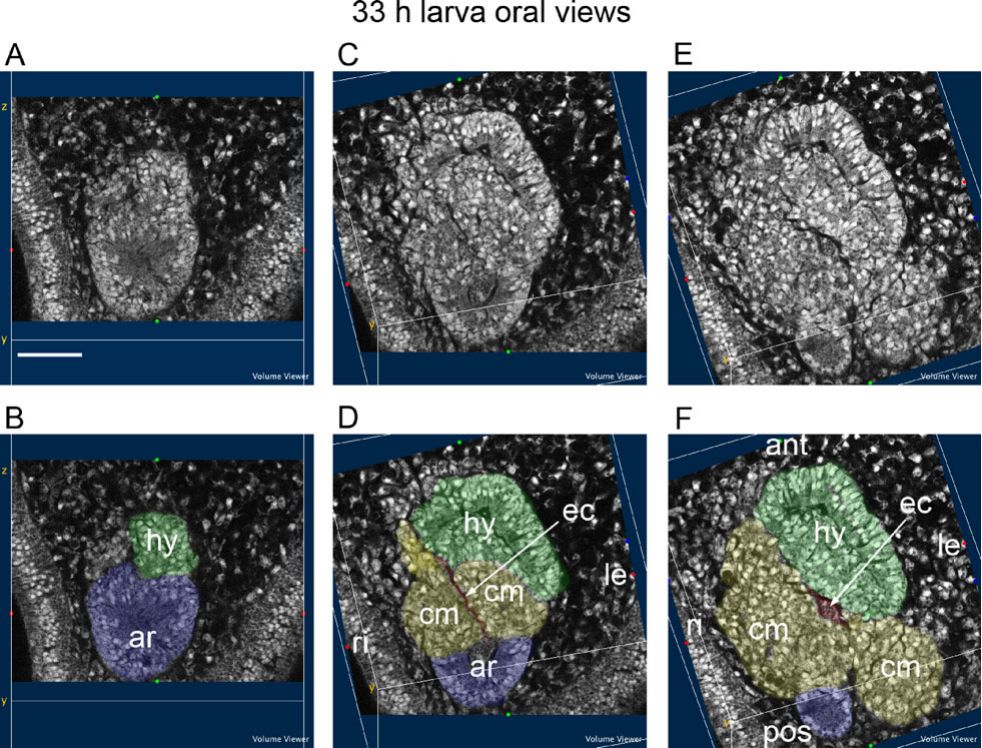
**Origin of the hydrocoele and the coelomic mesoderm from the archenteron in oral view.** 33 h larva progressing from aboral to oral. (A,C,E) Uncoloured sections. (B,D,F). The same sections coloured with tissues of the hydrocoele (hy) green, coelomic mesoderm (cm) yellow, enteric channel (ec) red and archenteron (ar) blue. ant, anterior; le, left; pos, posterior; ri, right. Scale bar: 50 µm.

**Fig. 4.**
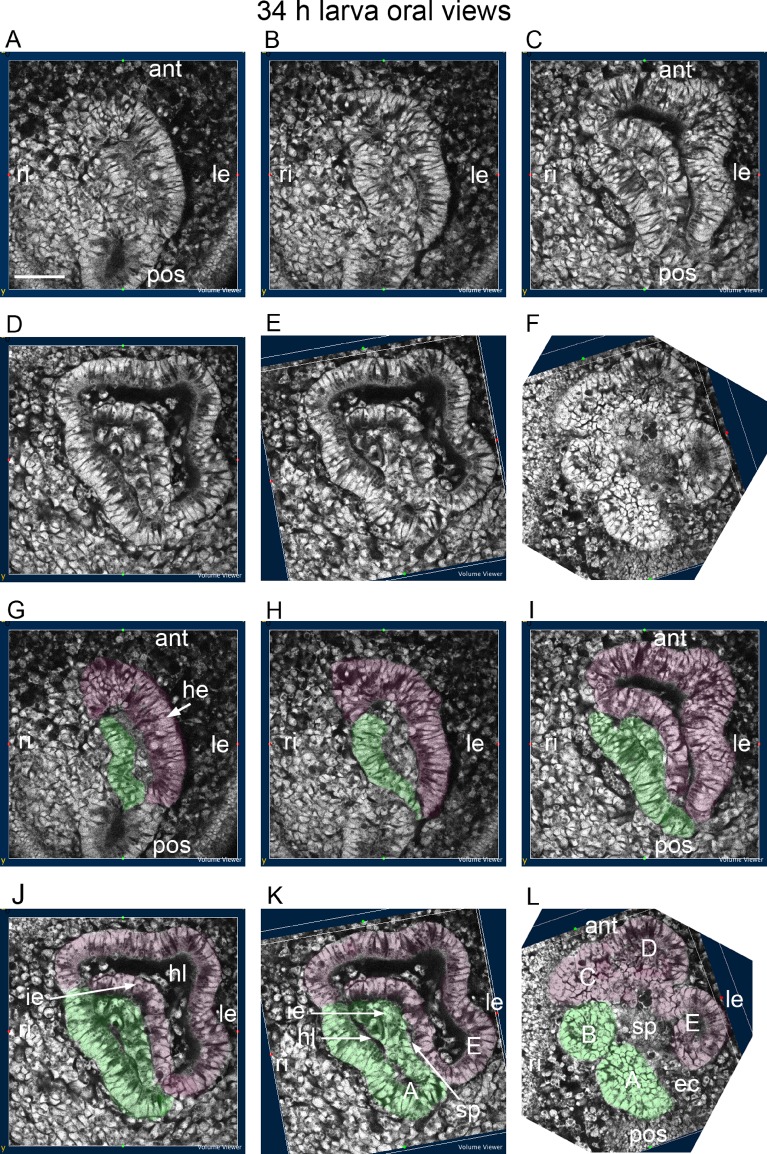
**Development of the hydrocoele and the origins of the primary podia.** Oral view of a 34 h larva progressing from aboral to oral. (A-F) Uncoloured sections showing the origin of the hydrocoele at the head of the archenteron and the different origins of the C, D and E podia and the A and B podia. (G-L) The same sections with the C,D,E epithelia and podia coloured magenta and the A and B epithelia and podia coloured green. ant, anterior; ec, enteric channel; he, hydrocoele epithelium; hl, hydrocoele lumen; ie, inner epithelium; le, left; pos, posterior; ri, right; sp, space between inner epithelia. The podia are labelled A,B,C,D,E. Scale bar: 50 µm. The *Z* stack from which this figure was constructed can be accessed at http://hdl.handle.net/2123/14231 at Sydney eScholarship Repository, The University of Sydney.

**Fig. 5.**
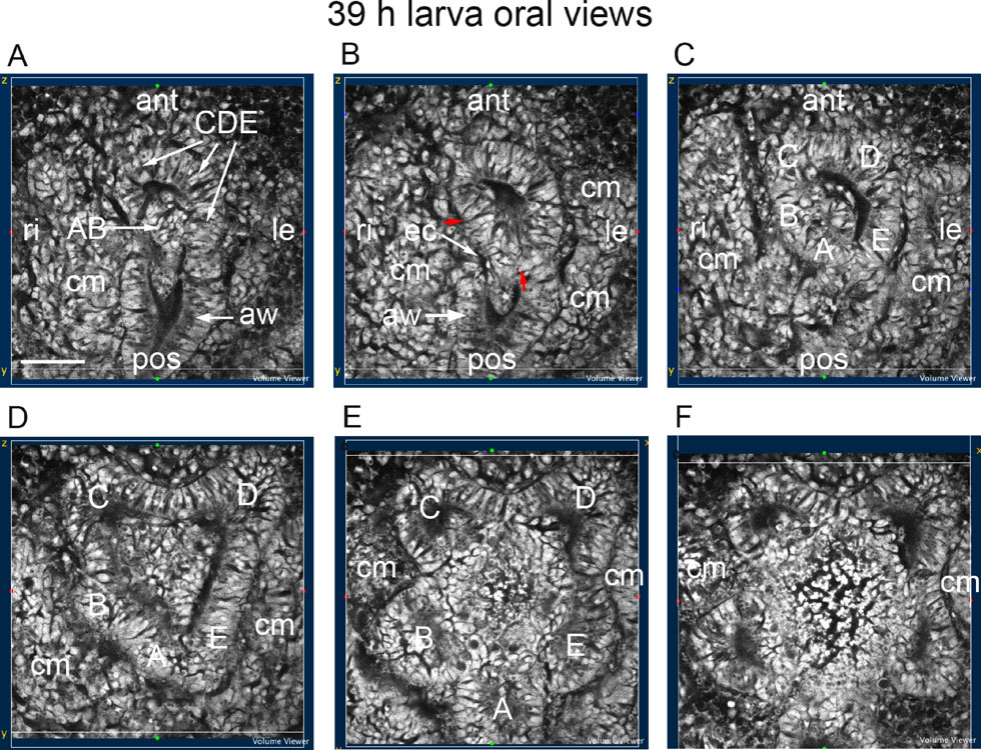
**Later development of the coeloms.** Oral view of a 39 h larva progressing from aboral to oral. (A,B) Epithelia of the C, D and E podia connect with the left anterior archenteron wall; epithelia of the A and B podia connect with archenteron tissue lying centrally; red arrows mark the boundaries between the AB epithelia and the CDE epithelia; coelomic mesoderm (cm) forms from both sides of the archenteron; the enteric channel (ec) is between the AB epithelia and the coelomic mesoderm forming on the right side of the archenteron. (C-F) The hydrocoele develops lobes (A, B, C, D, E) that will separate into the five primary podia. ant, anterior; aw, archenteron wall; le, left; pos, posterior; ri, right. Scale bar: 50 µm.

**Fig. 6.**
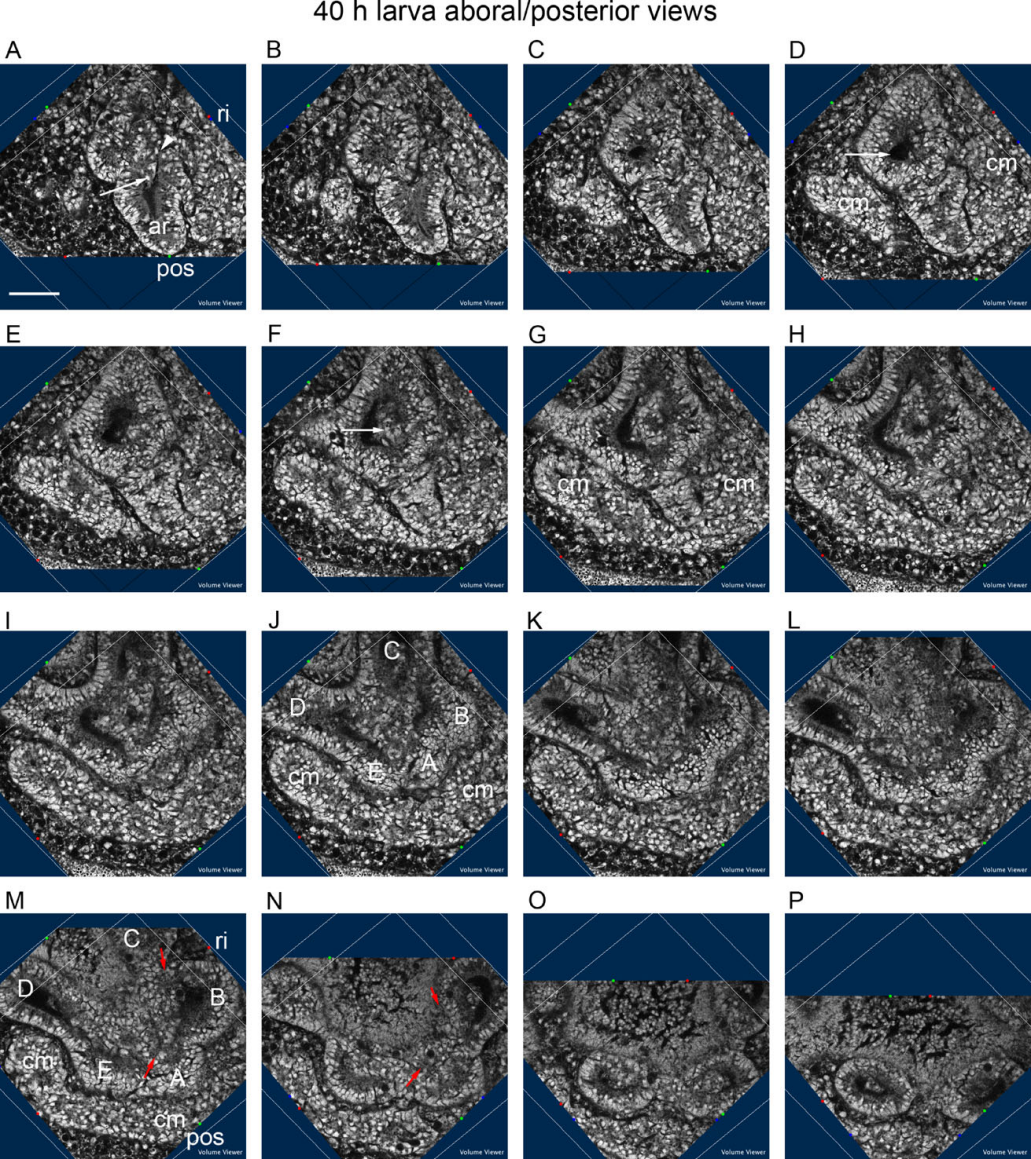
**Composition of the hydrocoele.** Sections progress along a *Z* axis slanted from aboral to oral and from posterior to anterior. (A-D) Aboral sections showing the source of the AB epithelia from a stem-like connexion (A, arrow) to the archenteron (ar); arrowhead in A points to the enteric channel; arrow in D points to the CDE lumen. (E-H) Tissue (F, arrow) spreads across the hydrocoele lumen. (I-L) The tissue spreads towards the outer epithelia of the podia. (M-P) AB podial tissue separates from CDE podial tissue along a line marked by red arrows; outer podial epithelia round in, separating the podia. (A-P) Coelomic mesoderm (cm) spreads orally about the hydrocoele. A, B, C, D, E label the five primary podia; pos, posterior; ri, right. 40 h larva. Scale bar: 50 µm.

**Fig. 7.**
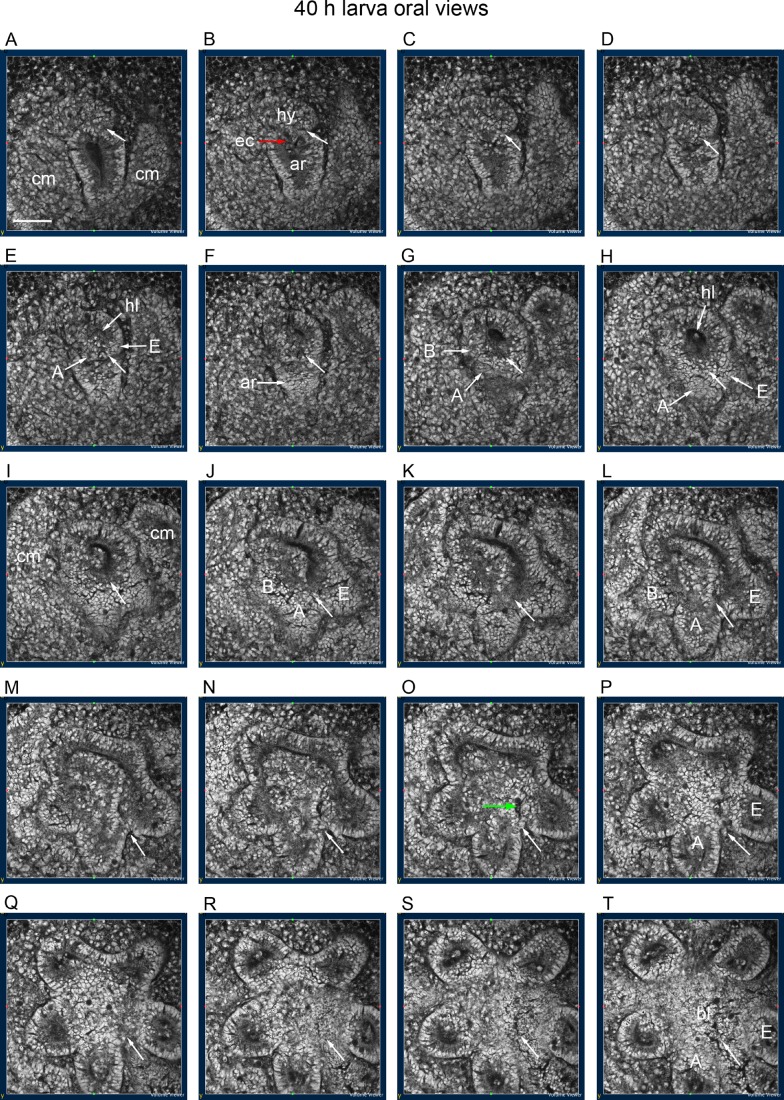
**Track of the enteric channel.** Oral view, sections progressing from aboral to oral. White arrows mark the boundary between the A and E epithelia throughout. (A-D) Enteric channel (ec, red arrow) starts at the base of a stem-like connexion of the hydrocoele (hy) to the archenteron (ar). (E-H) A and E epithelia grow over the archenteron; A and B epithelia become distinct. (I-L) Lobes develop from the A and E epithelia. (M-P) An opening develops between the epithelial boundaries of the A and E lobes (green arrow). (Q-T) The opening widens into the blastocoele (bl). (A-P) Coelomic mesoderm (cm) wraps about the archenteron, hydrocoele and hydrocoele lobes. 40 h larva. hl, hydrocoele lumen. Scale bar 50 µm. The *Z* stack from which this figure was constructed can be accessed at http://hdl.handle.net/2123/14231 at Sydney eScholarship Repository, The University of Sydney.

The results cover a period starting at the early development of the coeloms from the archenteron, progressing through the later development of the hydrocoele to the formation of the five primary podia and the advancing coelomic mesoderm. They are sections from confocal stacks of larvae in which the plane of section has at times been processed to show specific structures and relationships. An early larva of *H. purpurescens* at low magnification (Fig. 1) shows the structure of the larva and its orientation relative to adult echinoderm axes. High magnification sections of the posterior part of the larva where the coeloms develop from the archenteron follow (Figs 2-7).

### Early development of the coeloms

At 27 h in a sagittal view of an early larva (Fig. 2A,B,C), two regions of coelomic tissue have formed at the anterior end of the archenteron. The coelomic tissue on the aboral side will form the hydrocoele while that on the oral side will form the coelomic mesoderm. Between the hydrocoele and the coelomic mesoderm is the enteric channel, a cavity that is continuous with the archenteron cavity and the blastopore.

At 29 h also in sagittal view (Fig. 2D,E,F), the hydrocoele and the coelomic mesoderm have enlarged, the enteric channel remains, but together the hydrocoele, coelomic mesoderm and enteric channel have turned towards the oral side of the larva. An epithelium has formed on the aboral and anterior sides of the hydrocoele.

At 29 h in a slightly more advanced larva in sagittal view (Fig. 2G-I), there is further enlargement of the hydrocoele and the coelomic mesoderm and both have turned further to the oral side such that the enteric channel now has an approximate oral-aboral orientation. A small portion of coelomic mesoderm lies beneath the hydrocoele and above the enteric channel.

In an oral view of a 29 h larva (Fig. 2J-L), the hydrocoele is anterior, the coelomic mesoderm is posterior and the cavity of the enteric channel is between them. The coelomic mesoderm is partially divided into two regions beneath the single hydrocoele.

The hydrocoele, coelomic mesoderm and enteric channel constitute the oral coelom of the larva (Morris, 2012). This oral coelom is the equivalent of the left coelom of an echinoid pluteus larva that in the pluteus larva forms the adult rudiment (Pearse and Cameron, 1991).

At 33 h, a series of three sections from one larva progressing from aboral to oral (Fig. 3) shows the origins of the hydrocoele and the coelomic mesoderm from the archenteron in oral view. The aboral section (Fig. 3A,B) shows the origin of the hydrocoele from the aboral wall of the archenteron. The next section (Fig. 3C,D) shows the origins of the coelomic mesoderm from the lateral and oral walls of the archenteron. The coelomic mesoderm on the left side partly covers the origin of the hydrocoele. The final oral section (Fig. 3E,F) shows the anterior position of the hydrocoele relative to the more posterior, extensive spread of the coelomic mesoderm that originated from the lateral and oral walls of the archenteron. The coelomic mesoderm formed on the right side is more extensive than the coelomic mesoderm on the left. The hydrocoele sits over the coelomic mesoderm on the left side. The archenteron is posterior to the coelomic mesoderm. The enteric channel is beneath the hydrocoele and between the left and the right coelomic mesoderm (Fig. 3C,D). Orally, the channel becomes an opening between the hydrocoele and the coelomic mesoderm (Fig. 3E,F).

### Development of the hydrocoele

The origins of the hydrocoele from the archenteron and the early development of five primary podia from the hydrocoele are shown in a series of sections of one larva at 34 h in oral view, progressing from aboral to oral (Fig. 4). The most aboral section (Fig. 4A,G) shows the hydrocoele with a well-formed epithelium on the left side. In the next section (Fig. 4B,H), there is a well-formed epithelium on both sides of the hydrocoele. The epithelium on the left side has formed aborally from the anterior archenteron wall. The origin of the epithelium on the right side of the hydrocoele is less clear but it seems to originate from archenteron cells lying to the right of where the left epithelium forms, as well as more orally (Fig. 4A,G).

In more oral sections, the hydrocoele epithelium on the left, and now anterior sides (Fig. 4C,I), has developed an inner epithelium enclosing a hydrocoele lumen (Fig. 4D,J). The hydrocoele epithelium on the right has a partially formed inner epithelium and a hydrocoele lumen (Fig. 4E,K). A space forms between the two inner epithelia (Fig. 4E,K). In the most oral section (Fig. 4F,L), the space has enlarged separating the inner epithelia: the space is possibly part of the enteric channel. The outer epithelia have the shape of the primary podial lobes and have separated partially enclosing the tips of the lobes (Fig. 4F,L). The primary podia form on the oral face of the hydrocoele.

Primary podia C, D and E and primary podia A and B (Fig. 4F,L) thus form from different regions of the hydrocoele. The C, D and E group form from the hydrocoele epithelium on the left and anterior of the hydrocoele and the A and B group form from the hydrocoele epithelium on the right. A and B develop slightly later than C, D and E.

The two primary podia A and E, being from the different groups, form from epithelia on different sides of the hydrocoele, with A forming on the right side and E on the left (Fig. 4E,K). The epithelia from which they originate are posterior, lying next to the anterior archenteron wall (Fig. 4A,G). Orally, the epithelia turn inwards forming the A and E podia and opening a space between A and E that is possibly continuous with the enteric channel (Fig. 4E,K and F,L).

### Later development of the coeloms

At 39 h (Fig. 5), the structural relationships between the early coeloms, the archenteron and the enteric channel (Figs 2 and 3) are still evident. In the aboral sections of a larva in oral view (Fig. 5A,B), the hydrocoele epithelium of the C, D and E podia (CDE epithelia) connects with the left anterior archenteron wall. This anterior wall also connects with the coelomic mesoderm on the left side (Fig. 5B,C). The hydrocoele epithelium of the A and B podia (AB epithelia) connects with archenteron tissue emerging centrally and orally from the anterior archenteron wall (Fig. 5A,B,C). The enteric channel lies between the AB epithelia and the coelomic mesoderm connecting with the right side of the archenteron wall (Fig. 5B). In two oral sections (Fig. 5C,D), the CDE and AB epithelia take on podial shapes. In the two, final oral sections (Fig. 5E,F), the five podial lobes are separated centrally and between the lobes. The coelomic mesoderm lies to the sides of both the archenteron and the hydrocoele, and orally, inserts between the podial lobes (Fig. 5A-F).

### Composition of the hydrocoele

The right, posterior part of the hydrocoele is formed by the AB epithelia, the remainder is formed by the CDE epithelia (Fig. 6). The aboral sections (Fig. 6A-D) in an aboral/posterior view of a 40 h larva show the hydrocoele with a stem-like connexion (Fig. 6A, arrow) to the archenteron. The stem-like connexion is the source of the AB epithelia (see also Fig. 5A). A lumen, the CDE lumen, is in the hydrocoele (Fig. 6D, arrow). In the next sections (Fig. 6E-H), tissue spreads across the lumen (F, arrow). In the next sections (Fig. 6I-L), the tissue partly separates the podial lobes. This tissue forms the inner epithelia of the podial lobes (see also Fig. 4C,J,K). In the remaining sections (Fig. 6M-P), a separation appears along a line between the AB podia and the CDE podia, that is, from between podia A and E to between podia B and C (Fig. 6M,N, red arrows). The podial, outer epithelia turn inwards joining with the inner epithelia (Fig. 6N,O) and separating the podia (Fig. 6P). The A and B podia retain a position at the right, posterior part of the hydrocoele (Fig. 6M, see also Fig. 4L). Coelomic mesoderm partially envelops the hydrocoele (Fig. 6A-P).

The plane of section in Fig. 6 progresses from both aboral to oral and posterior to anterior such that the view progresses from the archenteron through the starts of the AB epithelia and the CDE epithelia, the latter with a lumen. Tissue spreads across the CDE lumen forming the inner floor of the hydrocoele between the AB podia and the CDE podia. The view supports the concept of the hydrocoele as a two-part structure, the CDE part and AB part.

### Tracing the enteric channel

The enteric channel lies beneath the hydrocoele, between the hydrocoele and the coelomic mesoderm (Figs 2 and 5). At one end it connects with the archenteron cavity and at the other it opens into the blastocoele.

The channel is assumed to be the anlage of the path of the gut, including orally the oesophagus and mouth opening. The enteric channel is traced in a 40 h larva in oral view progressing from aboral to oral (Fig. 7A-T). In the aboral sections (Fig. 7A-D), the enteric channel (Fig. 7B, red arrow) is at the base of a stem-like connexion between the hydrocoele and the archenteron. In the next sections (Fig. 7E-H), the A and E epithelia that are joined around a hydrocoele lumen obscure the connexion between the hydrocoele and the archenteron. The boundary between the A and E epithelia is marked by white arrows (Fig. 7A-H). In more oral sections (Fig. 7I-L), the A and E epithelia have formed lobes. In the next sections (Fig. 7M-P), the boundary between the A and E lobes (Fig. 7M-P, white arrows) is traced to an opening into the blastocoele between the epithelia of the A and E lobes (Fig. 7O, green arrow). In the last sections (Fig. 7Q-T), this opening widens into the blastocoele, possibly at the end of the enteric channel. Whether the lumina of the A and E lobes join around the opening (Fig. 7O) or whether only the epithelia of the lobes form a contact is not clear. Thus, whether this is a closure of the hydrocoele ring about the region of the enteric channel at this level is unclear.

## DISCUSSION

Aspects of coelom development in *H. purpurescens* following gastrulation are described from an analysis of sections of larvae recorded by confocal laser scanning microscopy. At the earliest times, the coelomic tissue is separated into hydrocoele and coelomic mesoderm. Between the hydrocoele and the coelomic mesoderm is the enteric channel, which connects with the cavity of the archenteron. The enteric channel persists throughout later development. The hydrocoele forms on the aboral side of the archenteron. The coelomic mesoderm forms on the lateral and oral sides of the archenteron. The development of the coelomic mesoderm is asymmetrical with a greater contribution from cells on the right side of the archenteron than on the left. There is a close association between the left coelomic mesoderm and the hydrocoele. The hydrocoele develops five primary podia that form as a group of three, the C, D and E podia and a group of two, the A and B podia. The relative positions at which the podia will form become apparent aborally as the hydrocoele starts to form lobes. The podia develop from the lobes orally next to the epithelium of the vestibule. The coelomic mesoderm spreads anteriorly and orally around the hydrocoele and into space between the hydrocoele lobes. The enteric channel and archenteron are assumed to be where the parts of the gut will form including orally the oesophagus and mouth. The further growth of the larva to the adult from growth zones perhaps at the oral bases of the primary podia is such that the first-formed secondary podia and plates of the arms will be nearest the mouth (Hyman, 1955).

**Fig. 8.**
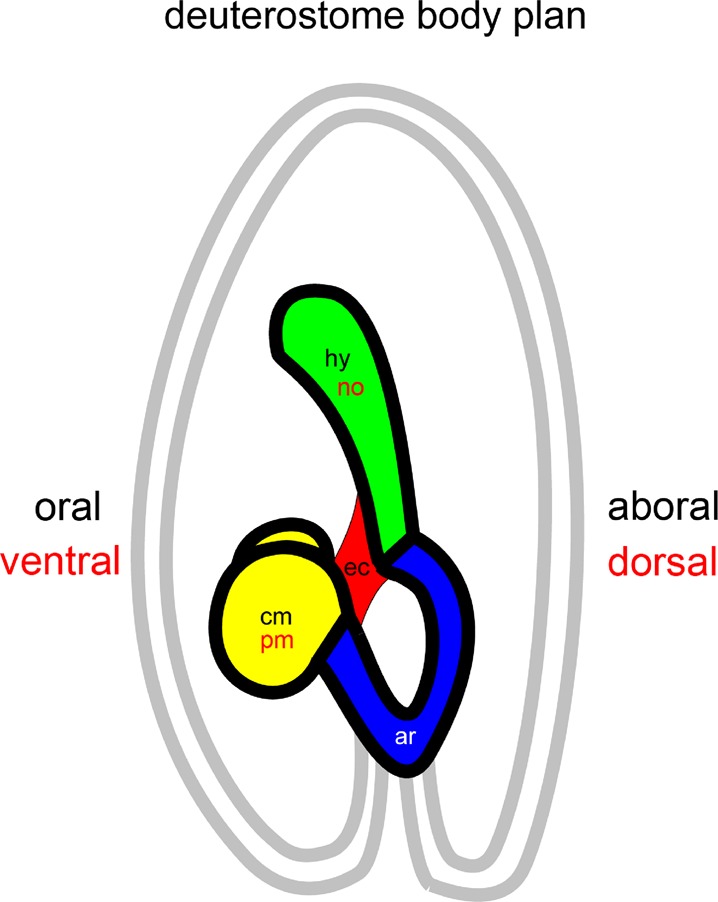
**The deuterostome body plan.** The plan is constructed from core structures of early coelom development in the larva of *H. purpurescens* and represents the form of the plan in an echinoderm. Red labels represent the plan in a vertebrate. ar, archenteron; cm, coelomic mesodem; ec, enteric channel; hy, hydrocoele; no, notochord; pm, paraxial mesoderm. Left-side sagittal view.

Core structures of the early coelom development can be schematized to derive a conceptual model of the body plan of deuterostomes (Fig. 8). The core structures are the hydrocoele coelom, the two lateral coeloms of coelomic mesoderm and the enteric channel. The relative positions of these structures are an essential part of the model. The medially-sited hydrocoele coelom lies over the enteric channel, which lies between the two lateral coeloms. The enteric channel connects with the archenteron. The close association between the hydrocoele and the left coelomic mesoderm may be added to the model, since it introduces the concept of a deuterostome body plan based on two coeloms, one incorporating the medial hydrocoele, allowing the deuterostome body plan to be derived from the two-coelom body plan of protostomes.

The deuterostome body plan derived from these core structures (Fig. 8) has a single axial or medial coelom overlying the enteric channel. To the sides of the enteric channel are a pair of coeloms. The enteric channel connects with the archenteron cavity. All three coeloms form from the archenteron.

The pentamery that develops from the hydrocoele is not included in the deuterostome body plan. The hydrocoele forms first as a single coelom and is treated as such in the plan. Pentamery, an echinoderm character, is not present in other deuterostomes and can be explained by duplication (Raff and Popodi, 1996; Morris, 2009, 2012) of the single ray structure defined by Jackson (see Mooi et al., 1994) of two columns of ambulacra plates bordered on each side by a single column of interambulacral plates. A single echinoderm arm or ray is thus the appropriate structure to use in comparisons between derivatives of the coeloms of deuterostomes. Duplication, as an explanation of pentamery, is to some extent supported by the way the CDE podia and the AB podia develop from the hydrocoele of the *H. purpurescens* larva.

Since a single arm or ray forms from a growth zone such that the older secondary podia and plates are nearer the mouth (Hyman, 1955), a posterior growth zone is included in the deuterostome body plan.

Applying the deuterostome body plan to echinoderms and chordates, the medial coelom is the hydrocoele in echinoderms and the notochord in chordates. The pair of coeloms in echinoderms is the coelomic mesoderm described here, and previously (Morris, 2012), and in chordates it is the paraxial and tail bud mesoderm. These coeloms form from the archenteron in echinoderms and chordates and the trajectory of metameric growth from a zone near the archenteron, older nearer the mouth, is the same in both, the metameric structures in echinoderms being the secondary podia and plates (Morris, 2012). Applying the plan to hemichordates, the inference is that the medial coelom is the proboscis coelom and the pair of coeloms is the coelomic tissue that forms on the sides of the archenteron (Kaul-Strehlow and Stach, 2013), possibly the coelomic tissue that forms in the collar with the metamerism attributed to the tentacles, which have a trajectory of growth, older nearer the mouth (Hyman, 1959, p176). The deuterostome body plan does not require the dorsoventral inversion of chordates, reviewed by Gerhart (2006).

### Support for the deuterostome body plan

Support for the proposed deuterostome body plan can be gained by considering how well it fits early frog and chick development, representing anamniote and amniote vertebrate development respectively (Gilbert, 2010). At gastrulation in a frog, involution at the dorsal lip of the blastopore is associated with the development of endomesoderm from which notochordal tissue separates medially. An endodermal layer underlies the notochordal tissue. Mesoderm forms laterally at the blastopore. The chick embryo forms a primitive streak whose opening is the blastopore (Bellairs and Osmond, 2005). The head process, the notochord, forms medially at the head of the primitive streak. Endoderm underlies the notochord. Mesoderm forms laterally from the sides of the primitive streak. Thus, both anamniote and amniote embryos broadly have the core structures of the deuterostome body plan of a medial coelom overlying an endodermal channel with coelomic tissue lateral to the endodermal channel.

**Fig. 9.**
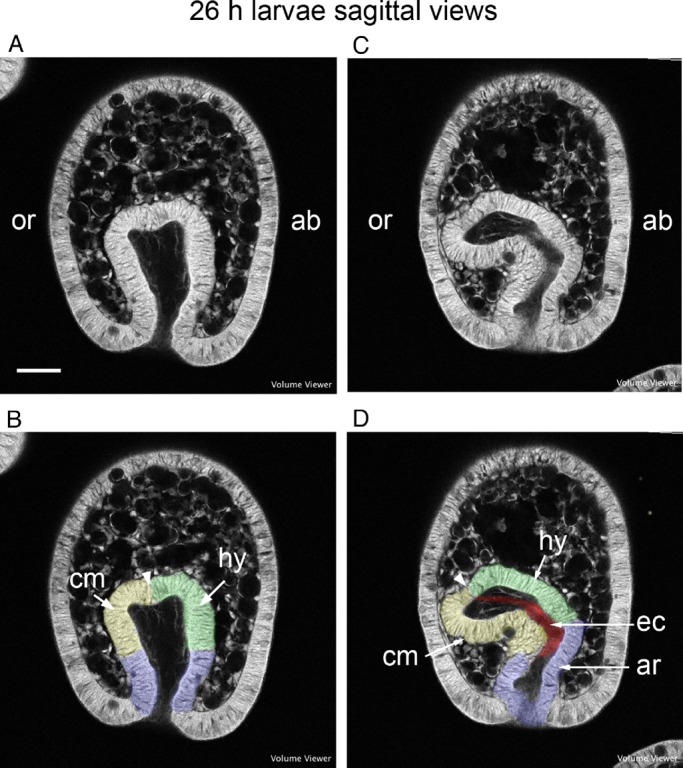
**Early coelom development in the larva of *Heliocidaris erythrogramma*.** (A,C) Uncoloured sections of 26 h larvae in sagittal views; A is slightly younger than C. (B,D) The same sections coloured with the putative anlagen of the hydrocoele (hy) green, coelomic mesoderm (cm) yellow, enteric channel (ec) red and archenteron (ar) blue. Arrowhead marks the notch between the hydrocoele and coelomic mesoderm anlagen. ab, aboral; or, oral. Scale bar: 50 µm.

Core structures of the deuterostome body plan can also be seen in the early development of the coeloms in the echinoid *Heliocidaris erythrogramma*, a species with no functional feeding larva and where, as in *H. purpurescens*, development leads directly to the juvenile sea urchin. Sections from confocal stacks of early development in *H. erythrogramma* reported on previously (Morris, 2011) show the following. At the earliest times, the coelomic tissue is separated into two regions (Fig. 9A,B) that can be likened to the early hydrocoele and coelomic mesoderm of *H. purpurescens* (Fig. 2A). A larval, left coelom develops, homologous with the oral coelom of *H. purpurescens*, with a lumen continuous with the archenteron cavity (Fig. 9C,D). The epithelium of the larval, left coelom can be divided into a region that will form the hydrocoele and one that will form the coelomic mesoderm (Fig. 9C,D). The putative enteric channel is between the hydrocoele and coelomic mesoderm (Fig. 9C,D). The arrangement of the hydrocoele, the coelomic mesoderm and the enteric channel in the gastrulae of *H. erythrogramma* (Fig. 9) resembles that in the gastrulae of *H. purpurescens* (cf. Fig. 9B,D with Fig. 2B,E). The difference between *H. erythrogramma* and *H. purpurescens* during early coelom development is that the epithelium of the larval, left coelom in *H. erythrogramma* is firmly structured around a lumen that is clear of cells, whereas the epithelium of the oral coelom of *H. purpurescens* is not well structured and the coelomic lumen contains cells. The separation into a hydrocoele with five primary podia and coelomic mesoderm appears later in *H. erythrogramma* than in *H. purpurescens*.

#### Relationship between direct and indirect development

Both *H. purpurescens* and *H. erythrogramma* develop directly, that is to say they do not form the functional feeding larva of sea urchins such as *Strongylocentrotus purpuratus* that develop indirectly. Whilst most sea urchins develop indirectly, direct development is not uncommon, having evolved independently from indirect developing forms at least 20 times (Wray, 1995). Although both forms of development lead to a homologous adult sea urchin as Love and Raff (2006) have pointed out, there is the question of whether the early coelom development in *H. purpurescens* and *H. erythrogramma* is representative of the coelom development that forms the adult in indirect development. That it is representative depends in part on showing that the larval coeloms in direct and indirect development are homologous. This was done by Emlet (1995) who identified the axes of the feeding larva from remnant feeding-larval spicules in the direct developing larva of *H. erythrogramma*. Homology between the oral coelom of *H. purpurescens* and the left coelom in indirect development cannot be demonstrated similarly from remnant feeding-larval spicules or a right coelom, since neither has been identified in *H. purpurescens*. The homology of the oral coelom and the left coelom is assumed here based on the outcome of both coeloms forming a homologous adult sea urchin and on the similarity described here between the development of the oral coelom of *H. purpurescens* and the left coelom of *H. erythrogramma*, given the homology established for *H. erythrogramma*.

The development of the left coelom into an adult rudiment in indirect development has not been described in the detail that it has in direct development. Even so, the early description of indirect development by von Ubisch (1913) and the recent description by Smith et al. (2008) show an anterior portion of the left coelom forming the hydrocoele and the posterior portion forming the left somatocoele, in much the same configuration as in the direct development of *H. purpurescens*, and of *H. erythrogramma* (Morris, 2011).

Recent data on gene expression in indirect development bear on the origins of the left coelom of the early pluteus larva. Luo and Su (2012) identify three domains of expression at the tip of the archenteron in the gastrula of *S. purpuratus*, an aboral domain, a roof domain and an oral domain. When the left and right coelomic pouches form, the gene expression in the pouches shows that while tissue from the roof and oral tip of the archenteron enter both pouches, the aboral tissue goes only to the left coelomic pouch. This aboral tissue later develops the hydroporic canal, a structure that joins the hydrocoele, so it is possible that the hydrocoele in indirect development derives from the aboral tip of the archenteron. Although the left coelomic pouch forms from different regions at the head of the archenteron, as does the oral coelom of *H. purpurescens*, the aboral tip of the archenteron in indirect development cannot be equated yet with the aboral coelom in the direct development of *H. purpurescens* from the morphological data presently available.

The external appearance of the pluteus larva of indirect development is highly modified compared with the larva of direct development, which has lost feeding structures, even so, the internal coeloms that form the adult might be conserved leading in both instances to a homologous adult sea urchin.

### Support from the fossil record

The interpretation of early coelom development in *H. purpurescens* offered here and the deuterostome body plan derived from it can be applied to data of the fossil record (Smith et al., 2013). The data show bilateral, trimeral and pentameral echinoderms co-existed during the Cambrian. It is suggested (Smith et al., 2013) that the first event in echinoderm evolution would have been the appearance of stereom plating. The capacity to form stereom plating might have evolved in paired, bilateral coelomic tissue in the echinoderm ancestor, tissue that was ancestral to the coelomic mesoderm described here. Such would account for the bilateral echinoderms. The next event would have been the appearance of an ambulacral-like food gathering structure over the mouth. This structure might have evolved from a third coelom that was the ancestral precursor of the axial or medial coelom described here. Duplication of this ambulacrum into three and then five would lead to the pentamery of echinoderms. The development of the podia as a group of three, the C, D and E podia and a group of two, the A and B podia, described here, is consistent with trimery and pentamery in the fossil data (Smith et al., 2013). The reduction of the plated region of a bilateral echinoderm as ambulacra increased in number to the pentameral structure of *Stromatocystites* (Smith et al., 2013) is consistent with the axial-extraxial theory of Mooi et al. (1994).

### Closure of the hydrocoele ring

With respect to closure of the hydrocoele ring, the interpretation here is that the AB podial group and the CDE podial group form from different regions of the anterior archenteron, that each has its own lumen and that the space between them connects with the enteric channel. Closure of the hydrocoele ring then requires that the lumina of the AB and CDE groups close, at some level, between A and E and also between B and C. Whether such closure occurs and the level where it occurs is not resolved by the present analysis. The matter is of importance because where the hydrocoele closes around the oesophagus has been described as differing between echinoids and asteroids. Traditionally, in the older literature, the closure is regarded as being between A and E in echinoids and between B and C in asteroids, as explained and discussed by Hotchkiss (1998).

### Conclusion

A deuterostome body plan has been derived from an analysis of early coelom development in *H. purpurescens* that is supported by observations of early coelom development in *H. erythrogramma*. It has been applied to examples of vertebrate development and to the echinoderm fossil record. It is expected that early development in deuterostomes will show evidence of three coeloms forming from the archenteron, with the third, medial coelom possibly forming in association with one of the other two coeloms. As a model of the structural homology of the deuterostome phyla, the plan contains the prediction that genes involved in coelomogenesis in creating a third coelom would have been those that initiated the macroevolutionary changes apparent in deuterostomes. Functional comparisons of genes involved in coelomogenesis in echinoids, amphibians and chick might contribute to understanding how such a macroevolutionary event came about.

## MATERIALS AND METHODS

Adult *H. purpurescens* were collected from coastal waters of New South Wales, Australia. Ova released from excised ovaries were fertilized with a diluted suspension of sperm from excised testes. Embryos and larvae from a fertilization were cultured as described (Morris, 1995) in filtered sea water (FSW) at 20 °C. Larvae were fixed at hourly intervals from 27 h to 40 h after fertilization for viewing by confocal laser scanning microscopy.

For the fixation, larvae were immersed in 4% (w/v) paraformaldehyde (Sigma-Aldrich, Castle Hill, Australia) in FSW for 2 h, washed in FSW, dehydrated in a series of methanols to 100% methanol (Sigma-Aldrich) and stored at −20 °C. For viewing in the microscope, larvae were cleared in 2:1 (v/v) methyl benzoate/methyl alcohol (Sigma-Aldrich) and mounted in the clearant in a cover-slip enclosed chamber set within a microscope slide.

Larvae were autofluorescent from the paraformaldehyde fixation. They were viewed in a Leica TCS SP5 MP multi-photon laser scanning confocal system (Leica Microsystems, Wetzlar, Germany) with a tunable Mai Tai Deep See laser (Spectra-Physics, Santa Clara, CA, USA) attached to a Leica DMI6000B-CS inverted microscope. Each specimen was imaged using multi-photon microscopy (Cox, 2007) at λ_ex_=870 nm with pulses in the 100-200 fs range and detected in a reflected non-descanned detector at λ_em_ 545-605 nm. A *Z*-stack was collected, with default *X* flipped, averaged over two frames in a 1024×1024 pixel array, 12 bits/pixel, at a slice thickness of 1.85 µm using a Leica HC PL APO 20×/0.70 IMM CORR CS objective lens or at a slice thickness of 0.5 µm using a Leica HCX PL APO 63×/1.30 GLYC CORR CS 21°C objective lens. The default *X* was flipped to restore the reflected image created in the inverted microscope to the non-reflected true image.

The *Z*-stacks were viewed in ImageJ (v. 1.43r). *XY* sections of the *Z*-stacks were supplemented by sections through any plane of the *Z*-stack created by the 3D plugin Volume Viewer.

The selected sections of larvae fixed at 27 h, 29 h, 33 h, 34 h, 39 h and 40 h, presented here, represent findings from the developmental period observed. Eight or more larvae were viewed from each of the fixation times.

### Terminology

The orientation of a larva in a section is described in relation to (1) the anterior-posterior (AP) axis in which the animal pole is anterior and the vegetal pole is posterior, and (2) the adult echinoderm oral-aboral axis, which is approximately orthogonal to the AP axis. The larva has a left and a right side defined as such in aboral view. In a sagittal view, the larva may be viewed from the left or the right side. These left and right sides are not those of the echinoid pluteus larva: the left side of the pluteus larva is the oral side of the *H. purpurescens* larva described here.

The five primary podia are labelled A, B, C, D and E after Hyman (1955, p40) following Cuénot (1891) see Morris (2007). The equivalent Lovén labels respective to the sequence above are V, I, II, III and IV.
